# Atypical Lead Pathway Leading to Vocal Cord Paralysis and Tracheostomy Following Pacemaker Implantation

**DOI:** 10.3390/jcm14134395

**Published:** 2025-06-20

**Authors:** Dariusz Jagielski, Jagoda Jacków-Nowicka, Bruno Hrymniak, Marek Kulbacki, Joanna Bladowska

**Affiliations:** 1 Faculty of Medicine, Wrocław University of Science and Technology, 50-370 Wroclaw, Poland; dariusz.jagielski@pwr.edu.pl (D.J.); marek.kulbacki@pwr.edu.pl (M.K.); joanna.bladowska@pwr.edu.pl (J.B.); 2 Department of Cardiology, Center for Heart Diseases, 4th Military Clinical Hospital, 50-981 Wroclaw, Poland; bruno.hrymniak@gmail.com; 3 Department of Radiology, 4th Military Clinical Hospital, 50-981 Wroclaw, Poland; 4 Faculty of Computer Science, Polish-Japanese Academy of Information Technology, 02-008 Warsaw, Poland

**Keywords:** pacemaker implantation, transvenous lead access, atypical lead pathway, venous anomalies

## Abstract

The axillary and cephalic veins are commonly utilized for transvenous pacemaker lead access. They typically advance to the heart through the subclavian, brachiocephalic, and superior vena cava veins. Anatomical variations such as a persistent left superior vena cava (PLSVC) may pose a challenge, necessitating an alternative approach for lead placement. This anomaly can often be identified during venographic contrast imaging or by visualizing atypical venous courses during the procedure. Another challenge occurs when the venous pathway is tortuous. Careful monitoring during the procedure is crucial to ensure that the lead follows the intended path. If not, the lead may inadvertently enter a collateral, such as the inferior thyroid vein, which drains into the internal jugular or left brachiocephalic vein. Despite these deviations, the lead may eventually reach the heart, although via an unusual course. If such a lead is left in place, even in the absence of immediate complications, long-term outcomes are unpredictable and carry the risk of unforeseen complications.

## 1. Introduction

**Figure 1:**
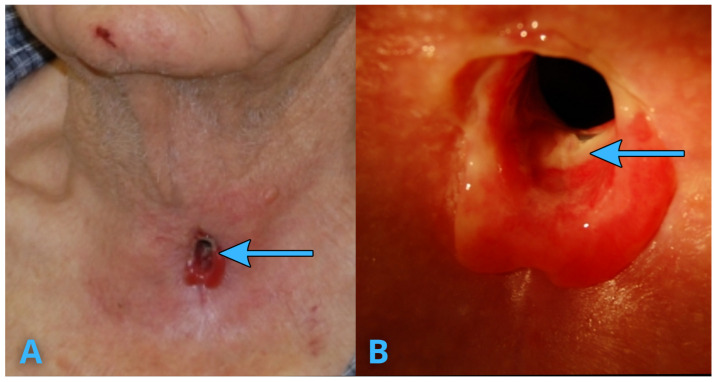
(**A**)—A photograph of the patient’s neck, (**B**)—A photograph in magnification. A lead fragment lodged in the tracheostomy wound. A physical examination revealed a fragment of the pacemaker lead (blue arrows) located in the lower part of the tracheostomy wound, indicative of lead migration.

We present the case of an 84-year-old male who had undergone a single-chamber atrial pacemaker (AAI) implantation for bradycardia associated with sick sinus syndrome. The initial implantation was uneventful, with no post-procedural complications related to the device pocket, stimulation parameters, or the infectious process. After 9 years, a routine device replacement was performed due to anticipated battery depletion. The procedure was uncomplicated, and post-replacement follow-ups confirmed proper lead function and device parameters without any procedural or early post-procedural complications. A year later, the patient developed unilateral and then bilateral vocal cord paralysis, leading to the need for a tracheostomy (Figure 1). Upon hospital admission, the ECG confirmed appropriate pacemaker stimulation in AAI mode. Echocardiography showed no signs of infective endocarditis. However, during physical examination, a fragment of the pacemaker lead was identified in the lower part of the tracheostomy wound. The patient was admitted to the cardiology department for further evaluation, and advanced imaging was performed to investigate the cause of the unusual lead positioning.

## 2. Imaging Studies

### 2.1. X-Ray Imaging

**Figure 2:**
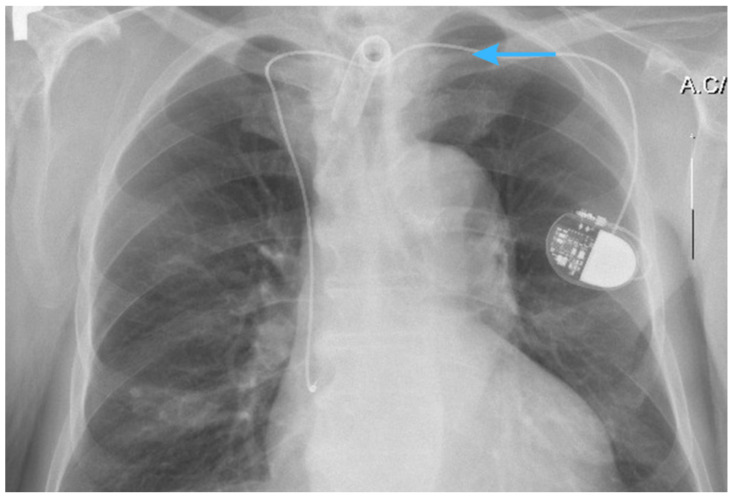
An atypical course of the pacemaker lead—chest X-ray illustrating an aberrant course of the pacemaker lead (blue arrow) traversing the upper mediastinum at the clavicular level and connecting to the pacemaker in the left middle lung field. The tracheostomy tube is centrally aligned within the upper mediastinum.

An initial chest X-ray examination revealed an atypical course of the pacemaker lead (Figure 2). The tip of the lead was visible in the typical place of the right atrium; however, it traversed unusually to the upper mediastinum at the level of the clavicles, connecting to the pacemaker in the projection of the left middle lung field. Furthermore, a centrally located tracheostomy tube at the level of the upper mediastinum was observed. Pneumothorax, inflammatory changes, and focal lesions within the lung fields were not observed, nor was there any fluid in the pleural cavities. There was an enlargement of the cardiac silhouette. To better understand the passage of the lead through the vascular system, additional imaging tests were ordered, including a CT scan.

### 2.2. Computed Tomography (CT) Scans of the Chest

**Figure 3:**
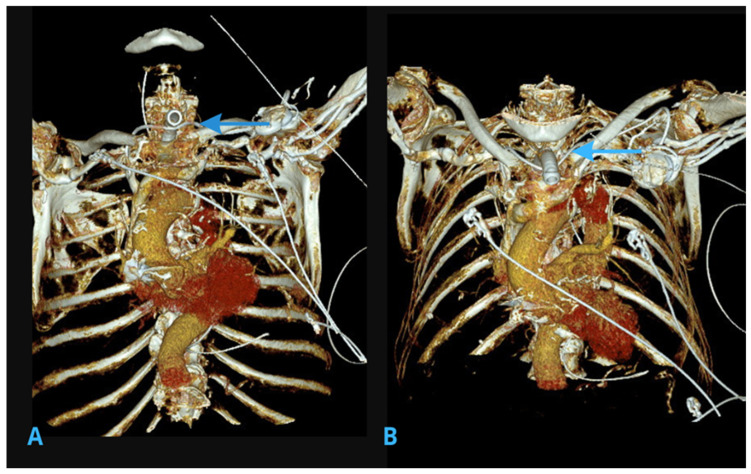
(Panel (**A**)) shows the inferior (bottom-up) view, while (panel (**B**)) presents the superior (top-down) perspective. A coronal computed tomography scan demonstrating the aberrant trajectory of the pacemaker lead through the inferior thyroid veins (highlighted by blue arrows), culminating in the lead fragment being positioned near the tracheostomy site.

**Figure 4:**
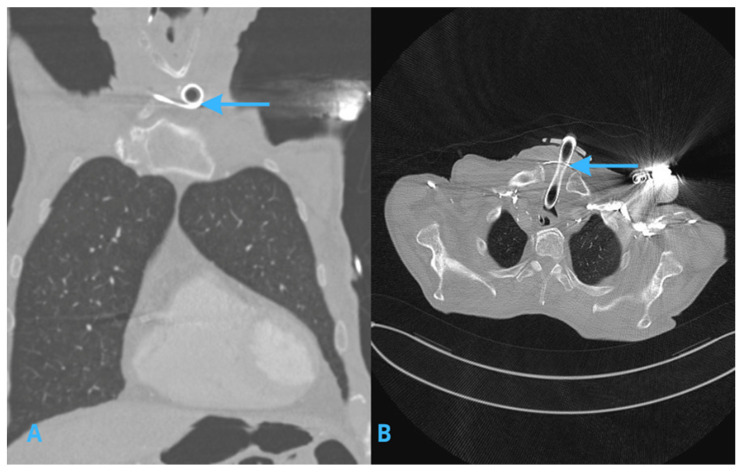
CT imaging of the chest, showing both coronal (**A**) and axial (**B**) cross-sections, illustrating the deviation of the pacemaker lead (blue arrows) into a collateral venous pathway, with the lead fragment positioned just below the tracheostomy tube. The image is displayed in the lung window for enhanced visualization of the lead’s trajectory.

CT scanning of the chest showed a precise view of the trajectory of the pacemaker lead (Figure 3 and Figure 4). The scans revealed that the lead had deviated from the typical venous path, moving through the left inferior thyroid vein, then the right inferior thyroid vein, and then finally entering the venous angle at the superior vena cava. These findings confirmed the suspicion, raised based on X-ray images, that the initial venous access had not followed the typical cephalic or subclavian vein, but rather a collateral venous route. Notably, this venous pathway culminated in the lead fragment being positioned at the tracheostomy wound’s lower part, explaining the observed complication. No other pathologies were identified in the chest CT scan.

## 3. Infection Risk and Management

Between imaging studies, a swab was collected from the tracheostomy wound, revealing colonization by *Staphylococcus lugdunensis*, a methicillin-resistant strain. The identification of this pathogen highlighted a critical aspect of the case—the presence of a potentially virulent and drug-resistant bacterium at the site of the implanted device. During an interdisciplinary consultation (vascular surgeon, cardiac surgeon, laryngologist, cardiologist–electrophysiologist, and echocardiographer), the patient was initially qualified for a procedure—transvenous extraction of the entire pacing system. After being informed of the risks associated with the proposed intervention, he declined to undergo the procedure and was discharged from hospital in a stable condition. However, in the following months, he developed sepsis, which ultimately led to his death. This underscores the importance of rigorous infection control practices and the early identification of potential sources of infection, particularly in patients with implanted devices [1,2]. AI-based monitoring systems could play an essential role in detecting early signs of infection or device malfunction.

## 4. Discussion

This case highlights an uncommon but significant complication of transvenous pacemaker implantation: an unusual venous pathway leading to bilateral vocal cord paralysis and, therefore, the need for a tracheostomy. Several key factors contributed to the complexity of this case, including the presence of an atypical venous anatomy, tortuous veins, and delayed recognition of the abnormal lead path. The integration of artificial intelligence (AI)-based systems for imaging analysis could have played a pivotal role in identifying the lead’s unusual course earlier in the procedure, potentially preventing the complications observed in this case [3]. As the literature describes, the early identification of lead deviations and their causes is crucial in preventing further complications, including nerve damage and migration-related issues [4].

### 4.1. Persistent Left Superior Vena Cava (PLSVC)

In our patient, the route taken by the pacemaker lead was consistent with a variation in venous anatomy, likely a PLSVC. This anomaly is a rare, congenital condition in which the left superior vena cava fails to regress during embryonic development, resulting in a left-sided venous return to the heart. As described in the literature, a PLSVC can be challenging to diagnose without advanced imaging, often necessitating a modified venous access strategy during pacemaker implantation [4]. Interestingly, this case did not show the classic findings of a PLSVC, but rather an aberrant route involving the inferior thyroid veins [4].

### 4.2. Collateral Venous Pathways and Tortuous Veins

Another consideration in this case was the patient’s advanced age and vascular changes that likely contributed to the tortuosity of the veins. Tortuosity of the venous system is commonly encountered in older adults and can complicate lead placement, as the lead may deviate into unexpected collateral veins [5]. In our patient, the left inferior thyroid vein served as an unintended route for the pacemaker lead. Subsequently, the lead followed a right-sided pathway through the right inferior thyroid vein before entering the superior vena cava. This abnormal lead course likely explains the subsequent complications, including vocal cord paralysis due to mechanical compression or the stimulation of the recurrent laryngeal nerve [6]. The tortuosity of veins and their anatomical variations in patients can present significant challenges in pacemaker implantation [4].

### 4.3. Lead Migration and Complications

The migration of pacemaker lead is a known complication, although it is more commonly associated with dislodgement or migration within the heart chambers. The migration of lead into a collateral venous pathway is an uncommon but documented phenomenon, and in this case, it resulted in the lead fragment becoming lodged in the tracheostomy wound, complicating the patient’s clinical course [7]. AI-enhanced imaging could improve the detection of lead migration or abnormal courses, allowing for timely intervention and potentially reducing the incidence of such complications [8]. Furthermore, emerging technologies such as leadless cardiac pacing offer the possibility of eliminating many complications associated with conventional transvenous systems, including lead migration, pneumothorax, pocket infections, and lead fractures. The wider implementation of leadless devices could significantly improve the long-term safety profile of cardiac pacing therapy.

### 4.4. A Practical Approach to Diagnostic Imaging

While X-ray imaging is a valuable first-line modality for identifying the abnormal positioning of pacemaker lead, it does not provide sufficient spatial resolution to fully see the anatomical courses of vascular structures. In everyday clinical practice, standard post-implantation chest X-ray remains essential for the first-line examination of pacemaker placement, helping with the rapid detection of malposition, detachment, or perforation. However, in cases where the trajectory of the lead appears atypical, advanced imaging becomes critical to prevent misinterpretation and to assess for potential complications, such as lead migration, vascular injury, or inadvertent entry into a non-target structure [1].

Multiplanar and three-dimensional CT reconstructions offer critical insights into the unexpected anatomical variations encountered during device implantation. This imaging modality not only enables the accurate localization of the lead but also provides essential information for potential revision procedures. Additionally, CT scans are invaluable in excluding other thoracic pathologies, such as vascular anomalies, masses, or iatrogenic injuries (for example pneumothorax, hematoma), which might coexist or result from complicated device placement. The integration of imaging findings with clinical presentation and procedural history ultimately supports safe and targeted patient management [1].

By offering a comprehensive view of the thoracic vasculature and surrounding structures, CT plays a pivotal role in managing complex cases of device misplacement, as well as possible complications, ensuring optimal outcomes [2].

### 4.5. AI-Assisted Imaging in Preventing Complications of CIED Implantation

Machine learning (ML) and AI have become groundbreaking technologies in cardiology, particularly in diagnostics and the prevention of complications associated with medical interventions [8]. Current AI systems, leveraging vast medical knowledge, process and analyze diverse patient data, including imaging from X-rays, CT, and MRI, with extraordinary precision [9]. As a result, AI-powered tools can detect problems earlier and facilitate more effective interventions. In the context of cardiac implantable electronic devices (CIEDs), AI holds significant potential for identifying anomalies, such as lead misplacement or migration, that could lead to serious complications. The clinical case presented herein underscores severe complications arising from improper lead placement and highlights the need for AI systems that can detect lead mis- or displacement early during follow-up imaging such as X-ray. The early identification of problems through AI-powered alerts could facilitate prompt corrections in lead positioning, preventing further complications and significantly improving patient outcomes [10].

AI systems designed for CIED monitoring must address challenges such as detecting improper lead placement, device migration, and structural interactions with adjacent anatomical features. By integrating imaging data with advanced AI algorithms, clinicians can gain access to tools that aid in the early identification of anomalies. Protocols like CaRDIA-X focus on manually identifying device patterns in X-ray images [11]. Such AI systems would require skillful training about the underlying artificial neural networks because datasets of rare cases may not be sufficiently large [12]. Moreover, automating these algorithms and integrating AI systems for the classification (such as anatomical and CIED localization) and optimization of CIED placement accuracy will improve precision and have the potential to enhance patient outcomes. This approach could significantly reduce the risk of complications such as lead misplacement, migration, and related cardiovascular events [13].

## 5. Conclusions

Herein, we report an exceptionally rare complication of permanent cardiac pacing, involving an atypical trajectory of the pacemaker lead that resulted in bilateral vocal cord paralysis and the need for tracheostomy. The clinical course was further complicated by sepsis, ultimately leading to a fatal outcome. This case underscores that late complications of transvenous pacing often pose a greater clinical challenge than early ones. Factors such as extensive fibrotic adhesions, increased risk of device-related infections, and progressive patient aging can elevate the procedural risk associated with transvenous lead extraction and contribute significantly to poorer outcomes. Although a wide range of complications can occur, device extraction has become a well-established and generally safe procedure in contemporary clinical practice [14].

Consequently, every effort must be made to ensure optimal lead placement during the initial implantation. Although chest X-ray examination is an easily available first-line imaging method, in the case of any suspicion of pathology including complications, CT of the chest should always be performed. Careful technique and thorough intraprocedural verification are essential to minimize the likelihood of complications that may only become apparent years later, when corrective interventions carry substantially greater risk. The use of advanced imaging techniques and artificial intelligence-based methods could have undoubtedly facilitated the early identification of the implantation error and its relatively straightforward correction, potentially preventing the severe complications observed in this case.

## Data Availability

Data are available from the corresponding author upon reasonable request.
